# Forensic Reconstruction of a Fatal Stair-Related Fall Using Postmortem CT, Photogrammetry, and Virtual Reality: A Case Report

**DOI:** 10.7759/cureus.81580

**Published:** 2025-04-01

**Authors:** Haruki Fukuda, Takayuki Nakazawa, Shoko Shinjo, Yoshihiko Kominato, Hiroyuki Tokue

**Affiliations:** 1 Department of Legal Medicine, Graduate School of Medicine, Gunma University, Maebashi, JPN; 2 Department of Diagnostic Forensic Science Laboratory, Gunma Prefectural Police Headquarters, Maebashi, JPN; 3 Department of Diagnostic and Interventional Radiology, Gunma University Hospital, Maebashi, JPN

**Keywords:** 3d documentation, autopsy, photogrammetry, postmortem computed tomography, staircase

## Abstract

Three-dimensional (3D) documentation is increasingly being utilized in forensic investigations to record injuries and reconstruct crime scenes accurately. Although photogrammetry offers a low-cost and accessible method for capturing surface details, its integration with postmortem CT (PMCT) data and virtual reality (VR) can further enhance spatial understanding. We report a fatal case of a stair-related fall of a man in his 70s, in which we visualized the injuries by combining PMCT data with surface models of the body and the staircase. A 3D model of the bones was created from the PMCT data, whereas surface models of the body and staircase were generated using photogrammetry. The reconstructed scene was visualized in VR using a MetaQuest 3 headset. The reconstructed scene clearly demonstrated the spatial relationship between the stair edges and injury sites, such as the occipital region, midthoracic spine, and sacrum. The vertical distances between the injuries closely matched the staircase step depth, supporting the interpretation of stair-related falls. This method provides an intuitive and immersive understanding of injury mechanisms. Our approach demonstrates the feasibility and utility of integrating CT, photogrammetry, and VR in forensic death investigations, offering enhanced documentation and visualization that can benefit not only forensic experts but also legal professionals and juries.

## Introduction

Three-dimensional (3D) documentation involves capturing and representing objects or scenes in three dimensions. It has demonstrated utility in forensic investigations by enabling accurate recording of forensic findings and allowing for subsequent review at any time [1-7]. This is achieved using various technologies, including photogrammetry, laser scanning, and structured light scanning for capturing external surfaces, as well as with CT for visualizing internal structures, enabling the detection of injuries such as fractures and intracranial hemorrhages [1,3]. Particularly, photogrammetry, which reconstructs 3D models by aligning multiple overlapping photographs taken from different angles and extracting spatial information through feature-matching algorithms, has been increasingly adopted owing to its simplicity, low cost, and ability to capture detailed surface features without specialized equipment [8]. Integrated 3D documentation that combines these technologies has been applied to the reconstruction of crime scenes and traffic accidents [4-7]. Furthermore, walkthroughs using virtual reality (VR) based on such 3D data have also been reported [9,10], which are useful not only for forensic experts but also for lawyers, judges, and members of the public who serve on juries by providing an intuitive and immersive understanding of complex scenes. However, integrated 3D documentation that combines CT data with surface information has only been reported in a limited number of facilities, partly because of the high cost of equipment and the complexity of the procedures involved.

In forensic death investigations, falling down stairs is a common injury mechanism that can lead to fatal outcomes [11]. Identifying injury patterns typical of stair-related falls and assessing whether abrasions and fractures correspond to the stair structure is crucial for evaluating whether a person found near a staircase actually fell and died due to the fall. These injuries are often described in two dimensions using autopsy sketches and measurements. To our knowledge, no previous reports have verified actual cases of stair-related falls by integrating postmortem CT (PMCT) data with surface information of both the body and scene.

Here, we report a fatal case of a stair-related fall in which 3D models of PMCT data, surface information of the body, and the actual scene were combined. Moreover, through visualization using a VR headset, the spatial relationship between the staircase, surface injuries, and fractures was clearly demonstrated.

## Case presentation

Case

A man in his 70s was found unconscious at the bottom of a staircase located in the first-floor corridor of an apartment building near his residence. He was transported to the hospital by emergency medical services, where cardiopulmonary resuscitation and right thoracic drainage were performed to treat the right-sided pneumothorax. Despite these efforts, death was pronounced approximately two hours after discovery. His medical history included coronary artery bypass grafting, aortic valve replacement, pacemaker implantation, and diabetes mellitus. The prescribed medications included clopidogrel, linagliptin (Tradjenta), nicorandil, and warfarin. The subsequent police investigation did not find any reason for his trespassing, nor did it suggest that he was involved in any crime.

PMCT findings

**Figure 1:**
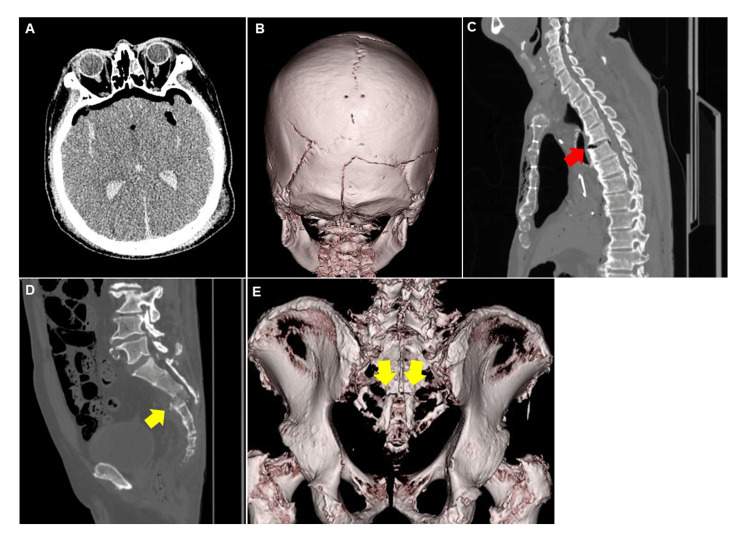
PMCT findings. (A) Axial image of the head. Pneumocephalus, subarachnoid hemorrhage, and intraventricular hemorrhage can be observed. (B) 3D volume-rendered image of the head. Fractures can be observed extending from the bilateral temporal bones to the occipital bone. (C) Sagittal image of the thoracic spine. Dislocation between the fourth and fifth thoracic vertebrae can be seen (red arrow). (D) Sagittal pelvic image. A sacral fracture can be observed (yellow arrow). (E) 3D volume-rendered image of the pelvis. A sacral fracture can be observed (yellow arrow).

Before the autopsy, PMCT was performed 16 hours postmortem using a 16-slice CT scanner, Alexion TSX-034A (Toshiba, Tokyo, Japan), with a slice thickness of 0.5 mm for the head (135 kV, 200 mAs, 1.5-second rotation) and 1 mm for the body (135 kV, 150 mAs, 1.0-second rotation) in the supine position. Bilateral fractures of the temporal and occipital bones were observed in the head, generally running in a transverse direction and extending to the cranial base (Figures 1A, 1B). Pneumocephalus, subarachnoid hemorrhage, and intraventricular hematoma were also observed (Figure 1A). In the thoracic region, a right-sided pneumothorax and multiple bilateral rib fractures were observed. In addition, separation between the fourth and fifth cervical vertebrae and a sacral fracture were identified (Figures 1C, 1D, 1E).

Autopsy findings

**Figure 2:**
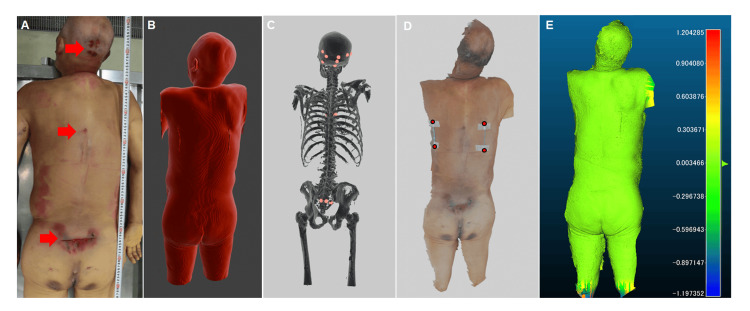
Photographs and 3D model of the body. (A) Photograph taken during the autopsy. Abrasion and subcutaneous hemorrhage can be observed in the head, midspine, and sacral regions. (B) Skin surface model reconstructed from postmortem CT (PMCT) data. (C) Bone model reconstructed from PMCT data. The red spheres indicate fracture locations. (D) Surface 3D model of the skin generated using photogrammetry. The catheter tubes were placed below the scapulae. Alignment between the PMCT model and the surface 3D model was performed using red markers. (E) Alignment analysis using CloudCompare. Green indicates a high degree of similarity between the two models, whereas red and blue indicate areas of greater discrepancy.

The patient was 167 cm tall and weighed 62.2 kg. External examination revealed abrasions and subcutaneous hemorrhages, primarily on the dorsal surface of the body. A 3.5-cm abrasion with subcutaneous hemorrhage was observed in the occipital region (157 cm above the soles of the feet). Additionally, a 2.5-cm abrasion with subcutaneous hemorrhage was noted in the mid-thoracic spine (126 cm above the soles), and an 11-cm transversely oriented abrasion with subcutaneous hemorrhage was found in the sacral region (91 cm above the soles) (Figure 2A). No other significant injuries were identified in addition to the signs of medical intervention.

On internal examination, consistent with the PMCT findings, a 26-cm-long fracture was observed extending through both the temporal and occipital bones, partially involving the cranial base. Traumatic subarachnoid hemorrhages were noted in the bilateral frontal, parietal, and temporal lobes, as well as on the posterior surface of the cerebellum. Additionally, a cerebral contusion was present in the lower medial region of the parietal lobe, along with bilateral uncal herniation. The dislocation between the T4 and T5 vertebrae, associated with surrounding epidural hemorrhages in the corresponding thoracic spinal cord, was identified in the torso. A 3-cm fracture was observed in the sacrum. Multiple rib fractures were observed bilaterally; however, some of these fractures showed only minimal hemorrhage and were considered attributable to cardiopulmonary resuscitation. No acute ischemic lesions were observed in the heart. Toxicological analysis of the urine yielded negative results. Pacemaker interrogation revealed no episodes of ventricular fibrillation after implantation. Based on these findings, we concluded that the individual died from intracranial injuries sustained after falling down a staircase and striking the occipital region and the dorsal surface of the body.

Creation of 3D models from postmortem CT data

After scanning the body in the supine position, PMCT was performed in the prone position under the same conditions as those before the autopsy, as the major injuries were located on the dorsal side of the body. Using OsiriX MD software [12], 3D models of the skin and skeletal structures were generated from the CT data and exported as OBJ files (Figures 2B, 2C). Two catheter tubes (11 cm in length) were affixed to the skin over the left and right scapular regions before the CT scan to facilitate alignment with the surface 3D model (Figure 2D).

Creation of 3D surface models of the body and staircase

To create surface 3D models via photogrammetry, photographs of the body in the supine position were taken from various angles using a Sony α6700 camera (image resolution: 6,192 × 4,128 pixels) following PMCT scanning and before autopsy. A total of 56 images were imported into RealityCapture software version 1.4.1.117424 (#Epic Games, Inc., Cary, NC, USA). With default settings, the images were aligned, and a mesh model was created, simplified using the “Simplify” tool, and textured. The 11-cm catheter tube served as a reference for scale adjustment. After trimming the unnecessary parts, the final model was exported as an OBJ file (Figure 2D). Similarly, a 3D staircase model was created using 144 photographs captured from the scene (image resolution: 6,192 × 4,128 pixels). The same photogrammetric workflow was applied to the scaled model based on a measuring tape placed at the scene.

Superimposition of 3D surface and CT-based models

The 3D surface model of the body and CT-derived skin and skeletal models were imported into the CloudCompare software. Automatic alignment was performed using the following four reference points: the upper and lower ends of the left and right catheter tubes, with the theoretical overlap set to 95%. The alignment yielded a root mean square error of 0.0265. According to the cloud-to-mesh distance calculation, the mean distance was -0.000864, and the standard deviation was 0.0901, indicating a high degree of spatial correspondence between the models (Figure 2E).

Scene reconstruction and visualization in virtual reality

**Figure 3:**
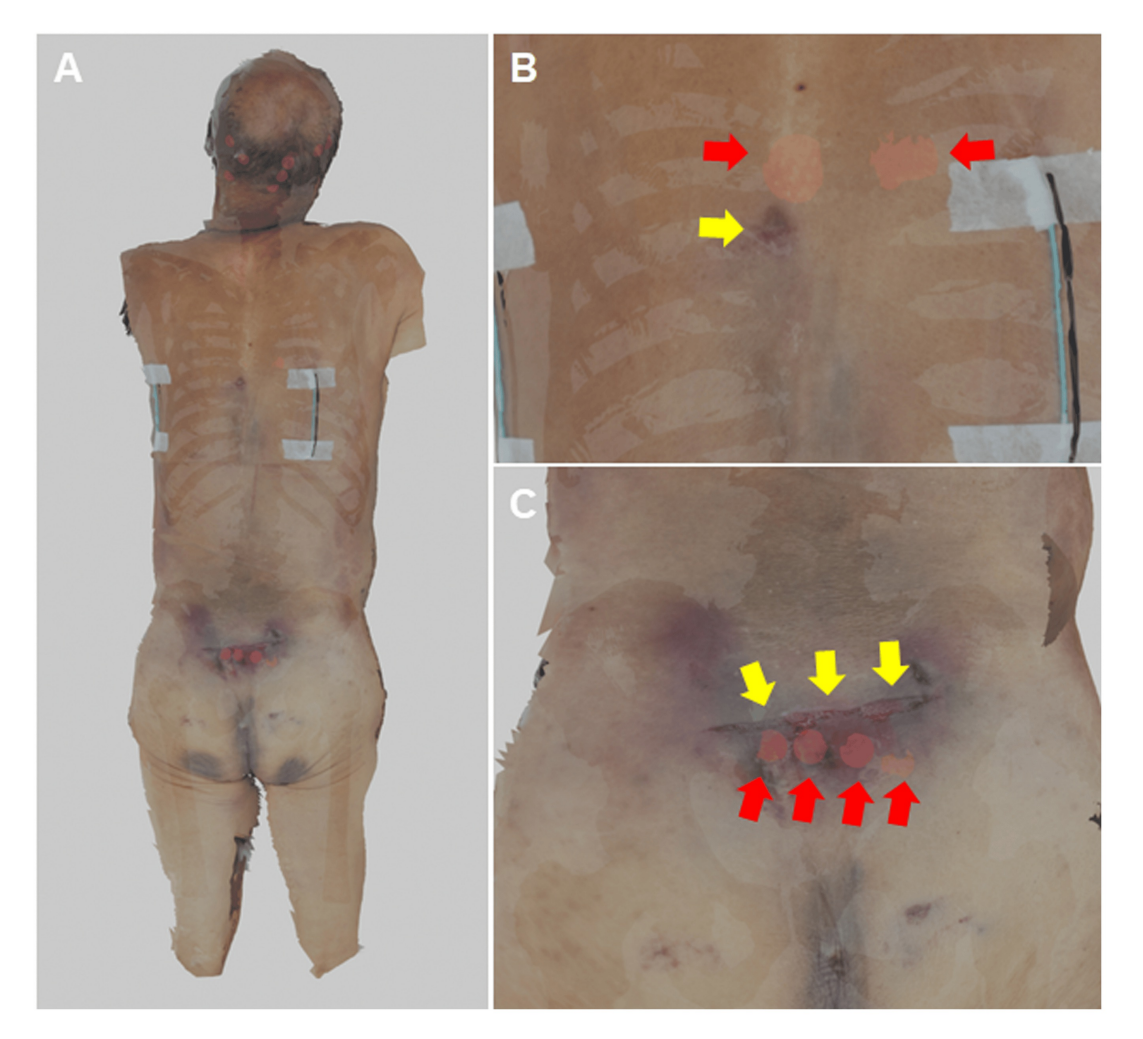
Combined visualization of postmortem CT and photogrammetry models. (A) Overview. The bones and red spheres indicate the fractures beneath the skin surface. (B) Enlarged view of the spinal region. (C) Enlarged view of the sacral region. Red spheres indicating fractures are visible, as indicated by red arrows. Yellow arrows indicate skin injuries.

**Figure 4:**
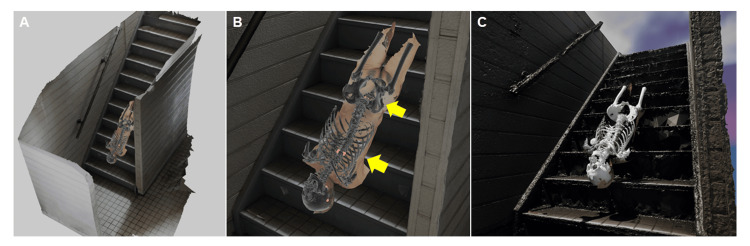
Scene reconstruction by integration of 3D models of postmortem CT data, surface information, and staircases. (A) Overall view. (B) Close-up view. As indicated by the yellow arrows, the width of the staircase corresponds approximately to the width of the body fractures. (C) Virtual reality-based scene reconstruction. For clarity, the bones are shown in white.

The 3D surface model of the body by photogrammetry, the PMCT-derived models of the skin and bones, and the 3D model of the staircase were imported into the Blender software [13] to reconstruct the scene. Red spheres were placed along the fracture sites in the 3D bone model to enhance the visibility of fracture lines. The skin model was set to “Alpha Blend” to render it partially transparent, and the bone model was assigned a black material to enhance visual contrast. The resulting scene was exported as an FBX file and imported into a customized project in Unreal Engine version 5.3.2 (© #Epic Games, Inc., Cary, NC, USA), based on the third-person template, with added VR functionality. Final visualization was performed using the MetaQuest 3 (Meta Platforms) headset. As a result, the spatial relationship between the stair edges and injuries on the body surface was clearly visible (Figures 3A-3C, Figures 4A-4C). The locations of the surface abrasions corresponded to the underlying fractures of the skull, thoracic vertebrae, and sacrum. Specifically, the vertical distance between the occipital abrasion (157 cm above the soles of the feet) and abrasion with subcutaneous hemorrhage in the midthoracic region (126 cm above the soles of the feet) was 31 cm, and the distance between the midthoracic injury (126 cm) and sacral abrasion with subcutaneous hemorrhage (91 cm above the soles of the feet) was 35 cm. These measurements closely matched the 32 cm step depth of the staircase where the body was found, supporting the interpretation that the injuries were consistent with a fall down the stairs. Additionally, the transverse orientation of both the skull fractures and the sacral injury aligned with impacts against the horizontal edges of the stairs, further supporting the fall-related injury mechanism.

## Discussion

In this report, we present a fatal fall case in which 3D models of PMCT data, surface information, and the actual scene were integrated, and the spatial relationships between the staircase, surface injuries, and skeletal fractures were clearly visualized using VR. Previous studies have shown that PMCT can effectively convey information that is difficult to understand in two-dimensional formats, especially for nonmedical professionals such as judges [14-16]. Furthermore, VR, with its immersive nature, enables enhanced visual comprehension and interactive engagement and is increasingly being applied in court presentations, crime scene reconstructions, and educational settings [10,17]. This case demonstrates that the combined use of PMCT, surface data, and scene reconstruction enhanced by immersive VR provides an intuitive and spatially accurate understanding of injury mechanisms in relation to the surrounding environment, an insight that might not have been fully captured using conventional two-dimensional documentation. Moreover, it holds potential as a cross-disciplinary tool that can bridge the gap between forensic medicine and other fields, such as legal practice and medical education.

Photogrammetry, which requires only a camera and software, offers a simple and cost-effective means of preserving 3D information without specialized equipment. The method presented here enables the seamless integration of CT and surface data, as well as VR-based visualization using relatively simple tools and procedures, making it a highly versatile and accessible approach.

However, this study has some limitations. Accurate alignment of CT and surface 3D data requires surface scanning to be performed with the body positioned identically to when the CT scan is taken. Although this was feasible at our facility, it may not be possible in all settings. In addition, when dorsal injuries are present, it is necessary to modify the standard supine CT scanning procedure to a prone position, which adds complexity and effort to the process.

Moreover, although enhancing the realism of 3D documentation is crucial for maintaining information accuracy, this may carry risks. As with autopsy photographs, highly realistic images can cause trauma to laypersons such as jurors, potentially complicating their admissibility as court materials. When using such data in legal proceedings, it may be necessary to apply visual modifications, such as converting images to grayscale, to preserve accuracy while reducing psychological impact. In the present case, 3D data of the scene were acquired at the actual location of the fall. However, the bloodstains had already been cleaned and thus did not reflect the state of the scene at the time of the incident. As the presence and characteristics of bloodstains can be critical in forensic investigations, preserving the original condition of the scene, including blood evidence, is desirable whenever possible.

In future applications, emerging techniques, such as Neural Radiance Fields, which enable 3D reconstruction from multiple photographs and are particularly effective for visualizing reflective and transparent surfaces, traditionally challenging for photogrammetry, have garnered attention. The integration of these methods with PMCT data for a comprehensive scene reconstruction is promising for forensic applications.

## Conclusions

We reconstructed a fatal stair-related fall by integrating PMCT data with surface models of the body and staircase and then visualized the injuries using VR. The spatial correspondence between the injury locations and the staircase structure supported the interpretation that the injuries were consistent with a stair-related fall. This method demonstrates how combining internal and external imaging techniques can enhance forensic interpretation, though alignment procedures may present practical limitations. The approach is cost-effective and promising for broader applications in forensic investigation, legal proceedings, and future research.
